# Reverse-Coded Items Do Not Work in Spanish: Data From Four Samples Using Established Measures

**DOI:** 10.3389/fpsyg.2022.828037

**Published:** 2022-06-23

**Authors:** Amanda Venta, Cassandra A. Bailey, Jesse Walker, Alfonso Mercado, Cecilia Colunga-Rodriguez, Mario Ángel-González, Gabriel Dávalos-Picazo

**Affiliations:** ^1^Department of Psychology, University of Houston, Houston, TX, United States; ^2^Department of Psychology, Sam Houston State University, Huntsville, TX, United States; ^3^Department of Psychological Sciences, University of Texas Rio Grande Valley, Edinburg, TX, United States; ^4^División de Ciencias de la Salud, Universidad de Guadalajara, Guadalajara, México; ^5^Centro Médico Nacional de Occidente, Instituto Mexicano del Seguro Social (IMSS), Guadalajara, México; ^6^Departamento de Psicología y Pedagogía, Universidad CEU San Pablo, Madrid, España

**Keywords:** Latino/a/x, reverse coding, reverse scored, reliability, Hispanic, translation

## Abstract

The potential for suboptimal psychometric performance of reverse-coded items may be particularly pronounced when scales are translated and administered in Spanish with these problems exacerbated in youth respondents. This is a significant concern, given the rapid rise in Hispanic-American and Spanish-speaking individuals in the US and their rightful, growing representation in psychological research and clinical care. The aim of this study was to examine the psychometric performance of reverse-coded items across four Spanish-speaking samples spanning developmental stages including youth, college students, and parents (*N* = 1,084; Adolescents *n* = 107; *M* = 19.79; *SD* = 2.09; 41.1% female; Caregivers *n* = 58; *M* = 40.79; *SD* = 7.94; 60.3% female; Spanish-speaking adults in the US *n* = 157; *M* = 33.4; *SD* = 9.5; 68.8% female; and College students living in Latin America *n* = 783; *M* = 21.04; *SD* = 3.13; 69.2% female) and four scales (Big Five Inventory; Strengths and Difficulties Questionnaire; Difficulties in Emotion Regulation Scale; Beck Hopelessness Scale); we expected reverse-coded items would demonstrate inadequate item–total correlations and their inclusion would compromise scale internal consistency. Hypotheses were supported with evidence of poor psychometric performance for at least two reverse-coded items on each instrument, such that un-reversing the items improved their item–total correlations. Across every instrument, alpha was either improved by excluding reverse-coded items or by including them in an un-reversed fashion and, overall, there was a moderate, negative effect of reverse-coded items on scale alphas. In growing consensus with previous authors, we recommend that reverse-coded items not be included in Spanish scales.

## Introduction

Spanish-speaking samples are growing increasingly represented in the psychological testing literature, as reflected by the establishment of the *Journal of Latinx Psychology* (formerly the *Journal of Latina/o Psychology*) in 2012 and special editorial sections related to research on translated instruments in journals like *Psychological Assessment* (e.g., Lima et al., 2017; Silva et al., 2018; García-Rubio et al., 2020). This increased focus on psychological assessment in Spanish mirrors growing awareness that ethnically and racially diverse samples are essential to generalizable psychometric research. Further, growth in the Hispanic-American population in the US has meant that practitioners and clinical scientists in the US are increasingly vested in psychological assessment with Hispanic-American individuals. Indeed, according to the US Census (2016), there were 40 million Americans speaking Spanish at home in 2016. All of these facts, together, require growing attention to the psychometric performance of published and translated instruments when conducted specifically with Hispanic-American samples in Spanish. The aim of this study was to evaluate empirical evidence for an anecdotal observation—that reverse-coded items do not work on the Spanish forms of well-established measures—utilizing data from four separate samples of Spanish-speaking, Hispanic-American respondents.

For quite some time, there have been both anecdotal reports and sporadic empirical reports that well-established measures, when translated to Spanish, pose psychometric problems when it comes to reverse-coded items—items that are worded (and scored) in a direction opposite from other items on the scale. For example, Salas-Wright et al. (2013), when examining a sample of Salvadoran adolescents completing the Basic Empathy Scale—a well-researched and widely used instrument, reported low item–total correlations (less than 0.3) for seven out of eight negatively phrased items on that scale, and noted that they were negatively correlated with positively worded items even after the scoring had been reversed. Further, the scale reliability, as measured by Cronbach’s alpha, was higher when using the reverse-coded items in a non-reversed manner (i.e., “this incorrectly coded analysis yielded a Cronbach alpha value of 0.795 as compared with Cronbach’s alpha value 0.659 for the 20-item scale that included the correctly recoded negative items” p. 1403). The authors concluded that reverse-coded items undermined the internal consistency of the scale. Similarly, in a sample of Spanish-speaking secondary school students, Sánchez-Carracedo et al. (2012) examined the factor structure of the Sociocultural Attitudes Towards Appearance Questionnaire and concluded that reverse-coded items should be excluded for improved scale performance. Galiana et al. (2016), analyzing the Spanish translation of the Balanced Measure of Psychological Needs in a large sample of Dominican adolescents, concluded that reverse-coded items negatively affected the scale’s factor structure, reliability, and validity.

Similar problems have also been identified with college students and adults. Guntzviller et al. (2011), in evaluating the Personal Report of Communication Apprehension-24 (McCroskey et al., 1985) among Spanish-speaking adults primarily from Mexico, found that reverse-coded and positive-coded items loaded onto separate factors. They speculated that translation to Spanish may have changed item meaning such that negatively worded items reflected a different construct than originally intended. Likewise, in development of their scale, the Foreign Language Anxiety in a Medical Office Scale, the negatively worded items factored separately from other scale items and were dropped from analyses. Regarding the Cognitive Test Anxiety Scale, Furlan et al. (2009) found, in a sample of Argentine Spanish-speaking college students, that reverse-coded items factored apart from other items and that scale performance was superior after eliminating reverse-coded items. Finally, in a sample of Spanish-speaking university students in Spain, Olivares et al. (2001) reported that reverse-coded items on the Spanish version of the Social Interaction Anxiety Scale possessed lower item–total correlations and loaded onto their own factor despite a theoretically unidimensional structure. In a particularly powerful study—an experimental study where the same Spanish participants were given a self-efficacy test with positive, reversed, and combined items, Suárez-Álvarez et al. (2018) reported that the inclusion of reversed items on the test negatively impacted its reliability and unidimensionality. While they note that including reverse-coded items may guard against acquiescence bias, they still conclude that their findings caution against use of reverse-coded items. Likewise, Vigil-Colet et al. (2020), analyzed two forms of the same test (with and without reverse-coded items) in a sample of Spanish college students while testing the effects of a procedure to control response bias effects. They reported that, when response biases were statistically controlled, reverse-coded items did not negatively impact the instrument’s psychometric properties but that, without this control, reverse-coded items should not be used.

It would be a mistake to imply that the debate about whether reverse-coded items weaken the psychometric properties of an instrument is about language of administration alone. Indeed, even when measures are administered in their original language of publication, like English, many researchers have commented that reverse-coded items generally had lower scale reliability (Weems and Onwuegbuzie, 2001; DiStefano and Motl, 2006) and it has been shown empirically for decades that reverse-coded items negatively impact reliability and validity (whereas negative keying of response options does not; Holden et al., 1985; Schriesheim et al., 1991). Reverse-coded items create wording effects—“systematic method variance caused by positive and negative item wordings on a self-report measure” (p. 142, Gu et al., 2015) that, unless modeled, affect scale reliability and validity and bias estimates (Gu et al., 2017). Indeed, in an experimental study conducted by Barnette (2000), the inclusion of reverse-coded items negatively impacted Cronbach’s alpha by 10–20%, leading the author to conclude that there are better ways to protect from acquiescence or response set behaviors, like changing the directionality of response options rather than item wording. However, these psychometric issues are exacerbated in individuals with low-reading comprehension (Williams and Swanson, 2001; Weems et al., 2006) and education levels (Benson and Wilcox, 1981), as well as among youth (Marsh, 1996), individuals from ethnic and racial minority groups (Schmitz and Baer, 2001), and when scales are used with international respondents (Nakano, 2001). Some authors have gone as far as to recommend that reverse-scored items not be used because linguistic translations of reverse-scored items are often undesirable and lead to poorer factor loadings (Sánchez-Carracedo et al., 2012).

Still, the aforementioned evidence of problematic psychometric properties related to reverse-coded items on Spanish language administrations is buried deep within psychometric analyses of individual measures. To our knowledge, there has not been a published report that primarily aimed to examine the performance of reverse-coded items among Spanish-speaking respondents. In all of the aforementioned research, the aim of the study was not to examine reverse-coded items or even scale internal consistency, but rather these analyses were reported in relation to other aims that often had little to do with measurement. We therefore sought to address this gap in the literature by providing empirical data on the psychometric performance of well-researched and widely used scales across four separate Spanish-speaking samples. This study is therefore the first in the published literature to draw data from multiple sources in order to authoritatively examine reverse-coded items on Spanish psychological assessments without focusing exclusively on one measure or idiosyncratic sample. Based on the aforementioned literature review and our own anecdotal experience collecting data from Spanish-speaking respondents, we expected that reverse-coded items would adversely affect the psychometric performance of the scales we examined as measured by Cronbach’s alpha, Omega reliability coefficient, and the scales’ internal structure.

## Materials and Methods

### Participants

#### Adolescent and Caregiver Samples

Participants were Spanish-speaking, recently immigrated undocumented high school students (*n* = 107) and their caregivers (*n* = 58; e.g., biological/foster parent, older sibling, aunt, uncle, cousin). All students were attending a Houston-area school and were originally from one of several Latin American countries (i.e., 16.4% Honduras, 26% El Salvador, 43.8% Guatemala, 8.2% Mexico, 2.7% Cuba, 1.4% another country in Central America, and 1.4% South America). High school participants ranged in age from 15 years old to 25 years old (*M* = 19.79; *SD* = 2.09), which encompasses the typical age range of public high school students in Texas (National Center for Education Statistics, 2015), while caregivers ranged in age from 23 to 63 years old (*M* = 40.79; *SD* = 7.94). The majority of the high school students were male (58.9%) and the modal number of years in the US was 2. Majority of caregivers were female (60.3%; 36.2% male; 1.7% transgender).

#### College Sample

Participants (*n* = 783) were young adults living in Latin America (born in: 77.7% Mexico, 18.9% Ecuador, 1.4% US, 0.9% Spain, 0.7% Peru, 0.2% Chile, 0.1% Morocco, and 0.1% Venezuela) and attending public universities there. All participants were 18 or older (*M* = 21.04; *SD* = 3.13) and were able to consent for themselves. Majority of college students were female (69.2%; 30.6% male; 0.1% transgender).

#### Adult Sample

Participants were non-detained, Spanish-speaking undocumented immigrant adults from Latin America (i.e., 3.2% Mexico, 12.7% Guatemala, 43.3% Honduras 18.5% El Salvador, 8.3% Venezuela, 13.4% Cuba, and 0.7% Dominican Republic) who were involved in removal proceedings at a Houston-area immigration court. A trained research assistant solicited participation face-to-face during Friday court screenings at the immigration court, through collaboration with several non-profit organizations. Participants were at court for the sole purpose of seeking legal services or to support a friend/family member who was seeking legal services. Participants ranged in age from 18 to 69 years old (*M* = 33.4; *SD* = 9.5). Out of the 157 participants, 31.2% were male (*n* = 49) and 68.8% were female (*n* = 108). Participants had been in the US for an average of 2.22 years (*SD* = 3.67; range = 0.01 to 22 years) and majority of participants (*n* = 111; 77.1%) had a high school education or less.

### Measures

#### Big Five Inventory

The Big Five Inventory (BFI) is an efficient and flexible assessment of the Big Five personality dimensions made up of 44 Likert scale items (of which 16 are reverse-coded) selected from Big Five prototype definitions (John, 1990). Items are rated from “strongly disagree” = 1 to “strongly agree” = 5. In support of the measure’s cross-cultural utility and cross-language validity, BFI items are short, allowing for easy translation into other languages (John et al., 1984; Hofstee, 1990; Benet-Martínez and John, 1998). Specifically, the Spanish self-report version of the BFI has demonstrated similar psychometric characteristics to English versions, with good internal consistency (Cronbach’s *α* = 0.77–0.82), consistent convergent and discriminant validity, replicative factor structure, and substantial construct validity with cross-language convergence across items (Benet-Martínez and John, 1998). This instrument was utilized in the adolescent sample.

#### Strengths and Difficulties Questionnaire

The Strengths and Difficulties Questionnaire (SDQ) is a long established assessment and screening measure of youth emotional–behavioral problems *via* youth, caregiver, or teacher report (Goodman, 1997). The SDQ’s 25 items (of which 5 are reverse-coded) are divided into five subscales with a total difficulties score determined by a sum of the first 20 items with higher total and subscale scores indicating higher emotional–behavioral problems. Items are rated from “not true” = 0 to “certainly true” = 2. Versions of the SDQ have generally shown adequate reliability as well as good criterion and convergent validity (Hill and Hughes, 2007; Tsang et al., 2012; Harry et al., 2019). Specifically, the Spanish caregiver version of the SDQ further demonstrates such strong efficacy (Gómez-Beneyto et al., 2013; Ortuño-Sierra et al., 2018; Harry et al., 2019), with reports of sufficient internal consistency reliability (McDonald’s ω = 0.84, Ordinal *α* = 0.75–0.81) in addition to similar factor structure and construct validity across studies utilizing other caregiver SDQ versions (Koskelainen et al., 2001; Percy et al., 2008; Ruchkin et al., 2008). This instrument was used in the caregiver sample.

#### Difficulties in Emotion Regulation Scale

The Difficulties in Emotion Regulation Scale (DERS) is a six-scale, 36-item self-report questionnaire (of which 11 are reverse-coded) created to assess general and specific aspects of emotion regulation difficulties (Gratz and Roemer, 2004). Items are rated “almost never” = 1 to “almost always” = 5. Although the DERS is a relatively young standardized measure, it has been translated across a number of languages with notable success (Cho, 2007; Ehring et al., 2008; Coutinho et al., 2010; Gómez-Simón et al., 2014). Respectively, the Spanish self-report version of the DERS has been shown to exhibit strong psychometric properties compared to both the original and other translated versions, with good internal consistency (Cronbach’s *α* = 0.71–0.84), convergent validity, and replicative factor structure with sufficient construct validity (Gómez-Simón et al., 2014; Wolz et al., 2015). This instrument was used in the college sample.

#### Beck Hopelessness Scale

The Beck Hopelessness Scale (BHS) is a self-report measure of one’s general tendency toward negative expectations about the future, consisting of 20 dichotomous items (of which 9 are reverse-coded; Beck et al., 1974). Items are rated “true” or “false.” The Spanish version of the BHS has been in use for roughly 25 years since its initial adaption (Aguilar García-Iturrospe et al., 1995) and has exhibited strong psychometric properties relative to the original measure. Specifically, the Spanish BHS has demonstrated strong internal consistency (Cronbach’s *α* = 0.82–0.84) and construct validity as well as moderate concurrent and predictive validity (Rueda-Jaimes et al., 2018; Satorres et al., 2018). While the original BHS was reported to have a three-factor solution consisting of “feelings about the future,” “loss of motivation,” and “future expectations” (Beck et al., 1974), more recent findings suggest the measure possesses a two-factor solution consisting of “self-referent negative expectation” and “generalized negative expectation” (Tanaka et al., 1998; Pompili et al., 2007; Nissim et al., 2010) of which the Spanish version of the BHS has supported (Satorres et al., 2018). This instrument was used in the adult sample.

### Procedures

#### General Procedures

All instruments used in this study were drawn from larger, archival datasets spanning three separate Institutional Review Board (IRB) approved studies (see below). Measures were not administered in order to test the effect of reverse-coded items on participants, rather, measures containing reverse-coded items were extracted after the completion of data collection in order to examine their psychometric performance.

#### Adolescent and Caregiver Sample

Institutional IRB approval (Sam Houston State University IRB-FY2016-26464) was obtained for this study as a part of a larger study of psychopathology, trauma, and migration experiences. To recruit participants, several trained research assistants visited every classroom of a Houston-area high school and orally explained the purpose, risks, and benefits of participating. Each student was given a consent form and letter explaining the study more in depth. This letter was to be given to their caregivers to sign or was signed themselves if over the age of 18. All students’ caregivers then received an automated phone call from the school explaining the purpose, risks, and benefits of the study. Classrooms were visited three times throughout the semester, and admission was rolling. Once consent was obtained, questionnaires were administered one-on-one to the student participants in Spanish. Because illiteracy rates are high in this population, items were read to each participant by a trained research assistant who was able to provide clarification to participants during the survey. On a separate occasion, within three months of the child being surveyed, caregivers were contacted *via* telephone to participate in caregiver report questionnaires. Once both the participant and caregiver completed their part of the study, the family was mailed a $20 gift card for their time (student participants were still compensated if their caregiver did not wish to participate themselves). Self-report packets were kept in a locked filing cabinet in a locked room.

#### College Sample

IRB approval was provided by the University of Texas Rio Grande Valley (IRB-18-0133). Data was collected online *via* Qualtrics from several international universities that are part of the *Red Cuerpos Academicos e Investigadores para el Desarrollo Humano Sustentable*. This international research group includes researchers from United States and multiple international universities in Mexico, Spain, Chile, and Ecuador. Appropriate IRB approval was obtained from participating collaborating institutions as part of a large-scale study of mental health symptoms among young adults left behind by parental migration. Participants were recruiting through their enrollment in specific courses, university list serves, and online research participation listings. Participation was completely voluntary and anonymous, and participants were able to decline participation at any time. Rather than collect signed informed consent, participants consented to participate after reading about the risks, benefits, and purpose of the study by clicking “accept” to advance to the questionnaire part of the study. The full study battery was administered in Spanish. For their participation, students were granted course extra credit.

#### Adult Sample

Prior to the commencement of data collection IRB approval was obtained (Sam Houston State University IRB-2018-13) as part of a larger dissertation study. Given that the main risk to participants is loss of confidentiality, a waiver of signed informed consent was acquired and no identifying information was obtained. Instead, consent to participate was obtained verbally after participants were explained the purpose, risks, and benefits of the study and provided a cover letter for their records. Participation took place at a Houston immigration courthouse. Once consent had been obtained, the questionnaire was administered one-on-one, in Spanish, by trained bilingual graduate and undergraduate students. The questionnaire utilized in this study was embedded in a larger assessment battery. Participants were assured that their responses would not affect their court proceedings in any way. Indeed, no assessment information was shared with attorneys, judges, or immigration officials. All participants (*n* = 157) were compensated with a $10 gift card for their time.

### Data Analytic Plan

Pairwise deletion was used to handle missing values. For each scale, item–total correlations were computed for each reverse-coded item. Those items were then un-reversed (e.g., a score of 2 on a 1–5 Likert scale would become a 4), yielding incorrectly scored items, and item–total correlations were again computed. For these analyses, item–total correlations greater than 0.2 were considered acceptable (Kline, 2015) though other conventions refer to 0.3 as the appropriate cutoff (Salas-Wright et al., 2013). Internal consistency was evaluated through Cronbach’s alphas and McDonald’s omegas computed for each scale including the reverse-coded items, excluding those items, and including them un-reversed (i.e., incorrectly scored such that an item that should be reverse-coded was instead left un-reversed, in the original response format). Alpha values greater than 0.70 were considered acceptable (Nunnally, 1994). Finally, across measures, a Pearson correlation was computed between the proportion of items on the scale that were reverse-coded and the alpha for that scale, in order to test the broad hypothesis that increased use of reverse-coded items would be associated with poorer internal consistency generally. Aforementioned analyses were completed using SPSS Statistics version 23. Measurement models were computed utilizing MPLUS version 8 (Muthén and Muthén, 1998–2017) in the two larger samples (Adult Sample, *n* = 157; College Sample, *n* = 783). Specifically, a measurement model was first specified with reverse-coded items and the factorial structure specified in the measure’s scoring instructions. Maximum likelihood estimation was utilized in examining a six-factor model for the DERS and diagonally weighted least squares estimation was utilized due to the dichotomous nature of BHS items. Second, the same model was specified excluding the reverse-coded items. Because the two models did not contain the same dependent variables, no formal measurement comparison could be undertaken but fit indices were reported and commented upon. Measurement models were examined as follows: DERS 6 factors and BHS 1 factor, as specified in each scale’s scoring instructions. Correlations between latent factors were freely estimated. In addition to *Χ*^2^, Root Mean Square Error (RMSEA), Comparative Fit Index (CFI), and Tucker–Lewis Index (TLI), and good fit was considered RMSEA value is less than 0.06 (Hu and Bentler, 1998), and CFI and TLI greater than 0.90 (Marsh et al., 2004).

## Results

### Adolescent Sample

Item–total correlations for each reverse-coded item on the BFI item are reported in Table 1. For this scale, subscale scores were used in item–total analyses given the scoring instructions for this instrument and the absence of a meaningful total score for the whole scale. Five out of 16 reverse-coded items demonstrated low item–total correlations when scored per the measure’s guidelines, affecting all scales other than Neuroticism. When those items were un-reversed, yielding incorrectly scored items, the performance of two items, as rated by item–total correlations, increased.

**Table 1:** Item–total correlations across measures and samples.

Measure	Sample	Reversed item	Item correlation with total score	Un-reversed item correlation with total
BFI	Adolescent	#6 Is reserved (E) *Es reservado*	0.185	0.123
		#21 Tends to be quiet (E) *Tiende a ser callado*	0.448	0.049
		#31 Is sometimes shy, inhibited (E) *Es a veces tímido, inhibido*	0.447	−0.016
		#2 Tends to find fault with others (A) *Tiende a ser criticón*	0.362	0.018
		#12 Starts quarrels with others (A) *Inicia disputas con los demás*	0.110	0.252
		#27 Can be cold and aloof (A) *Es a veces frío y distante*	0.409	0.125
		#37 Is sometimes rude to others (A) *Es a veces maleducado con los demás*	0.377	−0.075
		#8 Can be somewhat careless (C) *Puede a veces ser algo descuidado*	0.195	0.036
		#18 Tends to be disorganized (C) *Tiende a ser desorganizado*	0.441	−0.112
		#23 Tends to be lazy (C) *Tiende en ser flojo, vago*	0.327	−0.023
		#43 Is easily distracted (C) *Se distrae con facilidad*	0.322	−0.006
		#9 Is relaxed, handles stress well (N) *Es calmado, controla bien el estrés*	0.307	0.005
		#24 Is emotionally stable, not easily upset (N) *Es emocionalmente estable, difícil de alterar*	0.214	0.057
		#34 Remains calm in tense situations (N) *Mantiene la calma en situaciones difíciles*	0.303	0.141
		#35 Prefers work that is routine (O) *Prefiere trabajos rutinarios*	−0.040	0.074
		#41 Has few artistic interests (O) *Tiene pocos intereses artísticos*	0.119	−0.083
SDQ	Caregiver	#7 Generally well-behaved, usually does what adults request *Por lo general es obediente, suele hacer lo que le piden los adultos*	0.262	−0.155
		#11 Has at least one good friend *Tiene por lo menos un/a buen/a amigo/a*	0.212	−0.125
		#14 Generally liked by other youth *Por lo general cae bien a los otros niños/as*	0.013	0.198
		#21 Thinks things out before acting *Piensa las cosas antes de hacerlas*	0.247	−0.138
		#25 Good attention span, sees chores or homework through to the end *Termina lo que empieza, tiene buena concentración*	0.052	0.058
DERS	College	#1 I am clear about my feelings. *Tengo claro lo que siento*	0.367	0.018
		#2 I pay attention to how I feel *Pongo atención a cómo me siento*	0.391	0.026
		#6 I am attentive to my feelings *Estoy atento a mis sentimientos*	0.332	0.056
		#7 I know exactly how I am feeling *Sé exactamente cómo me estoy sintiendo*	0.434	−0.023
		#8 I care about what I am feeling *Le doy importancia a lo que estoy sintiendo*	0.262	0.116
		#10 When I’m upset, I acknowledge my emotions *Cuando estoy molesto, sé reconocer cuáles son mis emociones*	0.308	0.050
		#17 When I’m upset, I believe that my feelings are valid and important *Cuando estoy molesto, creo que ese sentimiento es lo adecuado y que es importante*	−0.287	0.408
		#20 When I’m upset, I can still get things done *Cuando estoy molesto, puedo conseguir hacer cosas igualmente*	0.234	0.014
		#22 When I’m upset, I know that I can find a way to eventually feel better *Cuando estoy molesto, sé que puedo encontrar alguna forma para conseguir finalmente sentirme mejor*	0.273	0.080
		#24 When I’m upset, I feel like I can remain in control of my behaviors *Cuando estoy molesto, creo que puedo controlar mi comportamiento*	0.317	−0.034
		#34 When I’m upset, I take time to figure out what I’m really feeling *Cuando estoy molesto, me doy un tiempo para comprender lo que estoy sintiendo realmente*	0.074	0.240
BHS	Adult	#1 I look forward to the future with hope and enthusiasm *Veo el futuro con esperanza y entusiasmo*	0.032	−0.025
		#3 When things are going badly, I am helped by knowing that they cannot stay that way forever *Cuando las cosas van mal pienso que no pueden quedarse asi siempre*	0.034	−0.009
		#5 I have enough time to accomplish the things I most want to do *Tengo tiempo para lograr las osas que más quiero hacer*	0.049	−0.010
		#6 In the future I expect to succeed in what concerns me most *En el futuro espero triunfar en las cosas que más me interesan*	0.021	−0.020
		#8 I happen to be particularly lucky and I expect to get more of the good things in life than the average person *Espero recibir más cosas buenas de la vida que la mayoría de las personas*	0.020	0.187
		#10 My past experiences have prepared me well for my future *Mis experiencias me han preparado para el futuro*	0.047	0.243
		#13 When I look ahead to the future I expect I will be happier than I am now *Cuando veo hacia el futuro tengo la esperanza de ser más feliz que ahora*	−0.005	−0.005
		#15 I have great faith in the future *Tengo fe n el futuro*	0.005	0.525
		#19 I can look forward to more good times than bad times *Puedo esperar más tiempos buenos que malos*	0.018	−0.021

BFI, Big five inventory; SDQ, Strengths and difficulties questionnaire; DERS, Difficulties in emotion regulation scale; BHS, Beck hopelessness scale; E, Extraversion; A, Agreeableness; C, Conscientiousness; N, Neuroticism; O, Openness.

For the BFI, internal consistency analyses relied on subscale analyses. Cronbach’s alpha for the Extraversion subscale including the reverse-coded items was 0.642, excluding those items was 0.582, and including those items but scoring them in a non-reverse-coded manner was 0.289. McDonald’s omega for the Extraversion subscale including the reverse-coded items was 0.637, excluding those items was 0.590, and including those items but scoring them in a non-reverse-coded manner was 0.037. Cronbach’s alpha for the Agreeableness subscale including the reverse-coded items was 0.647, excluding those items was 0.627, and including those items but scoring them in a non-reverse-coded manner was 0.308. McDonald’s omega for the Agreeableness subscale including the reverse-coded items was 0.596, excluding those items was 0.586, and including those items but scoring them in a non-reverse-coded manner was 0.135. Cronbach’s alpha for the Conscientiousness subscale including the reverse-coded items was 0.658, excluding those items was 0.580, and including those items but scoring them in a non-reverse-coded manner was 0.051. McDonald’s omega for the Conscientiousness subscale including the reverse-coded items was 0.651, excluding those items was 0.619, and including those items but scoring them in a non-reverse-coded manner was 0.093. Cronbach’s alpha for the Neuroticism subscale including the reverse-coded items was 0.580, excluding those items was 0.565, and including those items but scoring them in a non-reverse-coded manner was 0.357. McDonald’s omega for the Neuroticism subscale including the reverse-coded items was 0.586, excluding those items was 0.582, and including those items but scoring them in a non-reverse-coded manner was 0.196. Cronbach’s alpha for the Openness subscale including the reverse-coded items was 0.649, excluding those items was 0.732, and including those items but scoring them in a non-reverse-coded manner was 0.632. McDonald’s omega for the Openness subscale including the reverse-coded items was 0.654, excluding those items was 0.737, and including those items but scoring them in a non-reverse-coded manner was 0.670.

### Caregiver Subsample

Item–total correlations for each item on the SDQ are reported in Table 1. Out of five reverse-coded items, all of them displayed item–total correlations below 0.3, with two correlations falling below the 0.2 benchmark. In two instances, un-reversing the items yielded higher item–total correlations than scoring per the SDQ instructions.

Regarding internal consistency, Cronbach’s alpha including the reverse-coded items was 0.686, excluding those items was 0.707, and including those items but scoring them in a non-reverse-coded manner was 0.582. McDonald’s omega including the reverse-coded items was 0.271, excluding those items was 0.395, and including those items but scoring them in a non-reverse-coded manner was 0.314.

### College Student Sample

Item–total correlations for each reverse-coded item on the DERS are reported in Table 1. Two out of 11 reverse-coded items on that scale evidenced low item–total correlations and, in both instances, item–total correlations were improved to acceptable levels when the items were un-reversed and used incorrectly. Cronbach’s alpha for the DERS including the reverse-coded items was 0.929, excluding those items was 0.954, and including those items but scoring them in a non-reverse-coded manner was 0.906. McDonald’s omega for the DERS including the reverse-coded items was 0.930, excluding those items was 0.954, and including those items but scoring them in a non-reverse-coded manner was 0.907. The six-factor model including reverse-coded items demonstrated poor fit (*Χ*^2^ = 3944.98, *df* = 579, *p* < 0.001; RMSEA = 0.09; CFI = 0.80; TLI =0.79). When reverse-coded items were excluded, model fit was good with respect to some (*Χ*^2^ = 1622.51, *df* = 260, *p* < 0.001; CFI = 0.90; TLI =0.90) but not all (RMSEA = 0.08) fit statistics.

### Adult Sample

Item–total correlations for each reverse-coded item on the BHS are reported in Table 1. Correlations were below the 0.2 cutoff across the board, indicating that all nine reverse-coded items on this scale are problematic. In three instances, item–total correlations rose when the items were un-reversed, with the item–total correlations then falling in the acceptable range for two of the three items. Cronbach’s alpha including the reverse-coded items was 0.532, excluding those items was 0.480, and including those items but scoring them in a non-reverse-coded manner was 0.543. McDonald’s omega including the reverse-coded items was 0.627, excluding those items was 0.682, and including those items but scoring them in a non-reverse-coded manner was 0.611. The unidimensional model including reverse-coded items demonstrated poor fit (*Χ*^2^ = 339.24, *df* = 170, *p* < 0.001; RMSEA = 0.08; CFI = 0.71; TLI =0.68). When reverse-coded items were excluded, model fit was good (*Χ*^2^ = 66.84, *df* = 44, *p* = 0.015; RMSEA = 0.06; CFI = 0.91; TLI =0.89).

### Across Samples

In order to test the broad hypothesis that increased use of reverse-coded items would be associated with poorer internal consistency generally, the proportion of reverse-coded items on each scale and that scales alpha value were compiled in Table 2. The bivariate Pearson correlation computed between the proportion of items on the scale that were reverse-coded and the alpha for that scale was *r* = −0.361 (*p* = 0.380, *n* = 8) indicating a moderate, albeit non-significant, effect size demonstrating poorer internal consistency with a greater proportion of reverse-coded items.

**Table 2:** Proportion of reverse-coded items and alpha by scale.

Scale	Reversed items	Total items	Proportion of items reversed	Scale alpha
SDQ	5	25	0.20	0.69
DERS	11	36	0.31	0.93
BHS	9	20	0.45	0.53
BFI-E	3	8	0.38	0.64
BFI-A	4	9	0.44	0.65
BFI-C	4	9	0.44	0.66
BFI-N	3	8	0.38	0.58
BFI-O	2	10	0.20	0.65

SDQ, Strengths and difficulties questionnaire; DERS, Difficulties in emotion regulation scale; BHS, Beck hopelessness scale; BFI, Big five inventory; E, Extraversion; A, Agreeableness; C, Conscientiousness; N, Neuroticism; O, Openness.

## Discussion

The aim of this study was to examine the psychometric performance of reverse-coded items on well-researched and widely used scales across four separate Spanish-speaking samples. Based on a limited extant literature base, we expected that reverse-coded items would demonstrate inadequate item–total correlations and that their inclusion would compromise scale internal consistency and structure. On the whole, our hypotheses were supported with evidence of poor psychometric performance for at least two reverse-coded items on each instrument, multiple instances of reduced scale internal consistency, and poor model fit when including reverse-coded items. More specifically, across every instrument, alpha and omega were either improved by excluding reverse-coded items or by including them in an incorrect, un-reversed fashion. Likewise, for at least two items on every instrument, un-reversing the items (and using them incorrectly) improved their item–total correlations. Evidence of poor psychometric performance for reverse-coded items held in adolescent, caregiver, college student, and adult age groups and, further, across recent immigrants (adolescent and adult samples), immigrants living in the US for several years (caregivers), and non-immigrants living in Latin America (college students). Finally, bivariate analysis across measures indicated a negative relation between the proportion of reverse-coded items on a scale and its internal consistency, with a moderate, albeit non-significant, effect size.

Regarding the BFI, problematic item–total correlations affected five out of 16 reverse-coded items that appeared across all but one scale. Indeed, when those items were un-reversed and scored incorrectly, item–total correlations increased in two instances. Across the BFI scales, including reverse-coded items, correctly scored, made little difference. However, for the Openness scale, excluding reverse-coded items improved scale consistency and, further, un-reversing and including items was only marginally different from including them per the scoring instructions (alpha of 0.632 versus 0.649; omega 0.654 versus 0.670). In the caregiver sample, the SDQ demonstrated low item–total correlations for reverse-coded items across the board and, in two instances, the correlation actually increased when the items were un-reversed and used incorrectly. Likewise, Cronbach’s alpha and McDonald’s omega revealed an improvement in scale functioning when reverse-coded items were excluded and remarkably little difference when including them in an un-reversed, incorrect manner (alpha of 0.686 versus 0.582; omega 0.271 versus 0.314). Still, internal consistency estimates (both Cronbach’s alpha and McDonald’s omega) were low, indicating problems of reliability and echoing prior research calling into question the internal consistency and factor structure of the SDQ when administered in Spanish [(Author self-citation), Brown et al., 2014; Harry et al., 2019]. The DERS demonstrated fewer problems with reverse-coded items. Indeed, only two of 11 reverse-coded items showed problematic item–total correlations. However, both of those items performed better when un-reversed and, further, the scale’s alpha was improved when excluding reverse-coded items (alpha 0.954 versus 0.929; omega 0.954 versus 0.930). Model fit was good when reverse-coded items were excluded and poor when they were included. Regarding the BHS, all reverse-coded items demonstrated problematically low item–total correlations and, in three instances, incorrectly using the item by un-reversing it actually yielded a higher item–total correlation. Alpha calculations were also problematic, indicating that the scale reliability increased (from 0.532 to 0.543) when reverse-coded items were un-reversed and used incorrectly. Model fit was good when reverse-coded items were excluded and poor when they were included. Overall, every scale had at least some reverse-coded items that demonstrated unacceptable less than 0.2 item–total correlations and only the DERS displayed Cronbach’s alpha of at least 0.7 when including the reverse-coded items.

Consistent with the results of Salas-Wright et al. (2013) as well as Sánchez-Carracedo et al. (2012), our findings indicate poor performance of reverse-coded items when assessing adolescents in Spanish. Results from this study suggest that the problems identified by Salas-Wright et al. (2013) among Spanish-speaking adolescents in Latin America extend to adolescents who have migrated to the US and are attending school here, indicating that the psychometric problems identified both in this study and previously may be deleteriously affecting educational testing, psychological assessment, and research conducted with immigrant adolescents. Likewise, our findings echo those of Furlan et al. (2009); Guntzviller et al. (2011), and Olivares et al. (2001) by demonstrating problematic performance for reverse-coded items in Spanish-speaking college students again pointing to serious barriers for testing and research in this population. Still, it should be noted that reverse-coded items appeared to be less problematic in our college sample than in the other samples we assessed. While this difference may reflect idiosyncrasies of the samples, it may also lend support to the previously documented notion that reverse-coded item problems are exacerbated by education level (Benson and Wilcox, 1981). Indeed, the college student sample was, on average, the most highly educated sample included in our study. Still, the instrument utilized in that sample demonstrated psychometric problems with reverse-coded items nonetheless, suggesting that translation (Weems and Onwuegbuzie, 2001; DiStefano and Motl, 2006) and international data collection (Nakano, 2001) may have played a role, as in previous studies. Finally, it should be noted that reverse-coded items appeared highly problematic in both adult samples which, in this study, included adults awaiting immigration proceedings and adults caring for recently immigrated youth. Given that both of these subsamples are often included in psychological testing (e.g., for immigration hardship evaluations or as participants in educational or psychological testing for their dependents), our findings should raise alarm. Across both samples, low socio-economic status and education levels were the norm and, given data collection in the US, both samples were characterized as ethnic minority groups. All of these sample features have been previously identified as exacerbating psychometric problems on psychological scales (Benson and Wilcox, 1981; Schmitz and Baer, 2001; Williams and Swanson, 2001; Weems et al., 2006), and our findings support those previous results.

The current study is not without significant limitations. First, data were gathered from multiple, un-related data collection efforts in order to amass a collection of measures conducted with Spanish speakers in which the topic of reverse-coded items could be widely explored. Still, the instruments themselves, while all empirically validated and published in Spanish, vary in their psychometric performance regardless of translation and reverse-coded items. We did not control for this variability, as differential item functioning analyses between English and Spanish respondents would have been able to do. Nonetheless, the fact that we selected measures that were previously translated, had published psychometric data suggesting adequate performance, and are well-known instruments in the field of psychology and *still* documented that reverse-coded items decreased the psychometric performance of the scale is alarming. The good/adequate psychometric properties of these scales, as well as likely many others that were not included in the present analyses, may hide problematic reverse-coded items that take participants’ time and decrease the performance of the scale. Second, the samples themselves differ in important ways, and may have disproportionally included individuals with low-reading comprehension and educational attainment due to the overrepresentation of individuals of very low socio-economic status and migrants from poor, rural areas of Latin America (which have characterized Hispanic-American migrant flows to the Southwestern US in recent years). It is unfortunate that a standard Spanish literacy or IQ measure was not included in these archival datasets given that multiple samples have limited academic exposure. Spanish language, like English, varies among different cultures, places, and countries. While the samples included in this research are diverse with respect to nationality, it is important to examine subgroup differences in measure performance in the future, paying careful attention to culture, Spanish dialect variations, and indigenous language. Across the samples, the method of administration differed. While this may be seen as a confound in the current study, it also demonstrates that the problem of reverse-coded items exists regardless of how the measure is administered. Assessment of reverse-coded items on a brief desirability scale is important for future research. Third, analyses in the current study focused on scale reliability and internal structure and did not include analyses related to validity. While scale reliability is essential in placing an uppermost limit on scale validity, future research may endeavor to understand how the inclusion of reverse-coded items affects scale validity in Spanish. Fourth, the sample sizes for analyses differed considerably with one much larger (783) than the others (107, 58, and 157) and only two in which measurement models could be estimated. In most cases, the number of participants available to us was fewer than we would have hoped for psychometric evaluation. Still, these sample sizes are large when considering the available, published data on Spanish speakers, particularly vulnerable and unique samples of immigrants. Finally, our reliance on item–total correlations, Cronbach’s alpha, McDonald’s omega, and measurement model fit (in two samples) is limited in comparison to methods of analysis that are specifically designed to assess individual item performance (e.g., Item Response Theory). Still, using these accessible, common statistics allows for a birds-eye-view of how reverse-coded items perform in Spanish speakers across samples and instruments. Indeed, the varied ages, nationalities, and instruments represented in this study are a significant strength. In looking across our findings with four measures and more than 1,000 participants, we believe there is sufficient evidence that reverse-coded items impair scale performance in Spanish and that, as recommended by Sánchez-Carracedo et al. (2012), they should not be used.

## Author’s Note

Public Significance Statement: Across every instrument we investigated, reliability was either improved by excluding reverse-coded items or by including them in an incorrect, un-reversed fashion. Evidence of poor psychometric performance for reverse-coded items held in youth, caregiver, college student, and adult age groups and, further, across recent immigrants (adolescent and adult samples), immigrants living in the US for several years (caregivers), and non-immigrants living in Latin America (college students).

## Data Availability Statement

The raw data supporting the conclusions of this article will be made available by the authors, without undue reservation.

## Ethics Statement

The studies involving human participants were reviewed and approved by Sam Houston State University. Written, verbal, or implied (i.e., by way of advancing through the online survey) informed consent to participate in this study was provided by the participant or the participants’ legal guardian/next of kin when the participant was under the age of 18.

## Author Contributions

AV conceived of the study and conducted analyses. CB and JW supported analyses and writing. AM, CC-R, MG, and GP supported writing and data collection. All authors contributed to the article and approved the submitted version.

## Conflict of Interest

The authors declare that the research was conducted in the absence of any commercial or financial relationships that could be construed as a potential conflict of interest.

## Publisher’s Note

All claims expressed in this article are solely those of the authors and do not necessarily represent those of their affiliated organizations, or those of the publisher, the editors and the reviewers. Any product that may be evaluated in this article, or claim that may be made by its manufacturer, is not guaranteed or endorsed by the publisher.
